# Involvement of enhancer of zeste homolog 2 in cisplatin-resistance in ovarian cancer cells by interacting with several genes

**DOI:** 10.3892/mmr.2015.3745

**Published:** 2015-05-07

**Authors:** HUALI WANG, YUNHAI YU, CHEN CHEN, QIAN WANG, TAISHENG HUANG, FANGZHEN HONG, LIN ZHU

**Affiliations:** 1 Departments of Gynecology, The Second Hospital of Shandong University, Jinan, Shandong 250033, P.R. China; 2 Departments of Gynecology Obstetrics, The Second Hospital of Shandong University, Jinan, Shandong 250033, P.R. China

**Keywords:** ovarian cancer, gene expression data, differentially expressed genes, functional enrichment analysis, protein-protein interaction network, EZH2

## Abstract

In the present study, gene expression profiles of cisplatin-sensitive ovarian cancer (OC) cells were compared with those of cisplatin-resistant OC cells to identify key genes and pathways contributing to cisplatin resistance in ovarian cancer cells. The GSE15372 gene expression data set was downloaded from Gene Expression Omnibus, and included five biological replicates of cisplatin-sensitive OC cells and five biological replicates of cisplatin-resistant OC cells. Differentially expressed genes (DEGs) were screened using the limma package in *R*, based on the cut-off values of P<0.05 and |log_2_ (fold change)|>1. Kyoto Encyclopedia of Genes and Genomes pathway enrichment analysis and Gene Ontology enrichment analysis were performed on the DEGs using the Database for Annotation, Visualization and Integration Discovery. The protein-protein interaction (PPI) network was constructed for the DEGs using STRING, and sub-networks were analyzed by Clustering with Overlapping Neighborhood Expansion. A total of 556 DEGs were identified in the cisplatin-sensitive OC cells, of which 246 were upregulated and 310 were downregulated. Functional enrichment analysis revealed metabolism-associated pathways, DNA replication and cell cycle were significantly enriched in the downregulated genes, while cell growth and differentiation, response to stimulus, and apoptosis were significantly enriched in the upregulated genes. A PPI network, including 342 nodes was constructed for the DEGs and four subnetworks were extracted from the entire network. A total of 34 DEGs interacting with enhancer of zeste homolog 2 (EZH2) were identified, which were associated with DNA replication, pyrimidine metabolism and cell cycle. In conclusion, a number of key genes and pathways associated with the cisplatin-resistance of OC were revealed, particularly EZH2. These findings assist in the development of therapy for OC.

## Introduction

Ovarian cancer is the most life-threatening type of gynecological cancer, with a mortality rate of almost 14,000 in the United States alone in 2010 (1). The five-year survival rate for all stages of ovarian cancer is 47% (2). The poor prognosis results from the lacks of early detection or screening assessments, which leads to the majority of cases being undiagnosed until they have reached advanced stages.

Platinum-based cancer chemotherapy has been the general treatment approach for ovarian cancer for decades (3). However, >80% of patients eventually relapse with fully chemoresistant disease (4). The antitumor activity of cisplatin is based upon DNA damage via the formation of cisplatin-DNA adducts (5). The accumulation of DNA lesions can lead to steric obstruction of DNA-binding proteins, which are necessary for vital intracellular functions, and recognition of the lesions by high mobility group and mismatch repair proteins eventually lead to p53-initiated apoptosis (6–8). In addition, activation of the endoplasmic reticulum stress pathway also causes activation of apoptotic caspases (9).

Reduced drug uptake, decreased binding of cisplatin to DNA, DNA repair, decreased mismatch repair and impaired apoptosis have been considered as potential molecular mechanisms responsible for the platinum-based drug resistance (10–12). Lee *et al* observed that activation of the phosphoinositide 3-kinase/Akt pathway by phosphatase and tensin homolog reduction contributed to cisplatin resistance in an ovarian cancer cell line (13). Yang *et al* indicated that Akt leads to resistance via modulation of the action of p53 on the caspase-dependent mitochondrial death pathway (14). Li *et al* examined epigenetic changes and reported that DNA methylation is key in chemoresistance in ovarian cancer (15).

To further investigate altered gene expression profiles and relevant biological pathways, the present study performed a global and comparative analysis of the gene expression data between cisplatin-resistant ovarian cancer cells and cisplatin-sensitive ovarian cancer cells using bioinformatic tools, including functional enrichment analysis and protein-protein interaction (PPI) network analysis. The findings may advance current understanding of the molecular mechanisms underlying cisplatin resistance, and thus benefit the development of more effective approaches in the treatment of ovarian cancer.

## Materials and methods

### Gene expression data

The gene expression data (accession no. GSE15372) were downloaded from the Gene Expression Omnibus (http://www.ncbi.nlm.nih.gov/geo/), and included five biological replicates of cisplatin-sensitive A2780 epithelial ovarian cancer cells and five biological replicates of cisplatin-resistant Round5 A2780 epithelial ovarian cancer cells (Table I). The gene expression profiles were acquired using the Affymetrix Human Genome U133 Plus 2.0 array (Affymetrix Inc., Santa Clara, California, USA).

### Pre-treatment of raw data and differential analysis

The raw data in CEL format were read using the affy package in *R* (http://www.r-project.org) (16). Normalization was performed using a Robust Multi-array which consisted of three steps: Background adjustment, quantile normalization, and summarization (17). Gene expression values were averaged to calculate the final expression value for multiple probes corresponding to the same gene symbols. mRNAs, which were not detected in all samples were removed using the Affymetrix Microarray Suite 5 calls (MAS5CALLS) algorithm (Affymetrix, Inc.).

Differential analysis was performed using the limma package in *R* (18). P<0.05 and |log_2_ (fold change)|>1 were set as the cut-off values to screen out the differentially expressed genes (DEGs).

### Functional enrichment analysis

To determine the biological pathways altered in cisplatin-resistant ovarian cancer, Kyoto Encyclopedia of Genes and Genomes (KEGG) pathway and Gene Ontology (GO) enrichment analyses were performed on the DEGs using Database for Annotation, Visualization and Integration Discovery (DAVID; http://david.abcc.ncifcrf.gov/) (19). P<0.05 was set as the cut-off value.

### Construction of the protein-protein interaction (PPI) network

The PPI network was constructed for the DEGs using information provided by the Search Tool for the Retrieval of Interacting Genes (STRING) (http://string-db.org/) (20), and was subsequently visualized using Cytoscape (http://cytoscape.org) (21). Interactions with a score >0.4 were retained in the network. Proteins in the network served as the ‘nodes’, and each pairwise protein interaction, referred to as an ‘edge’, was presented as an undirected link. The sub-networks were then analyzed by Clustering with Overlapping Neighborhood Expansion (ClusterONE) (http://www.paccanarolab.org/clusterone) (22).

## Results

### Differentially expressed genes

A total of 69,954 transcripts were obtained from the raw data using the affy package and annotation files. Following removal of blank transcripts using the MAS5CALLS algorithm, 47,643 transcripts with expression levels were retained, from which 1,887 differentially expressed transcripts were identified in the cisplatin-sensitive ovarian cancer cells, including 815 upregulated transcripts, corresponding to 246 genes, and 1,072 downregulated transcripts, corresponding to 310 genes.

### Functional enrichment analysis results

**Figure 1:**
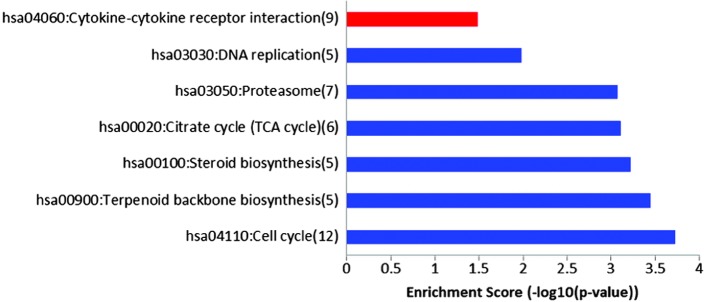
KEGG pathways significantly enriched in the differentially expressed genes. The KEGG pathways enriched in upregulated genes are indicated in red, while those enriched in downregulated genes are indicated in blue. Numbers in brackets indicate the gene number enriched in each corresponding pathway. KEGG, Kyoto Encyclopedia of Genes and Genomes.

**Figure 2:**
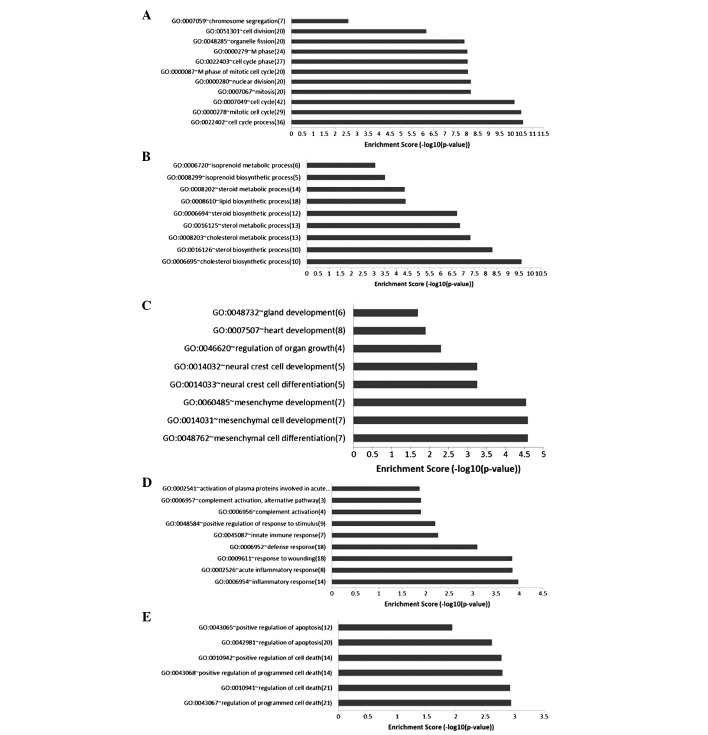
GO biological pathway terms significantly enriched in the differentially expressed genes. Downregulated genes in (A) cluster 1 and (B) cluster 2. Upregulated genes in (C) cluster 1, (D) cluster 2 and (E) cluster 3. GO, Gene Ontology.

The KEGG pathway enrichment analysis revealed that the metabolism-associated pathways, hsa00900 (terpenoid backbone biosynthesis), hsa00100 (steroid biosynthesis), hsa00020 (citrate cycle), hsa03030 (DNA replication) and hsa04110 (cell cycle) were enriched in the downregulated genes (Fig. 1). These pathways were associated with cell proliferation, which was inhibited by drugs in the cisplatin-sensitive cells. A total of 118 significant GO biological pathway terms were identified in the downregulated genes, which were divided into 12 clusters, of which two were associated with the cell cycle and metabolic process (Fig. 2).

Only one significant KEGG pathway was identified in the upregulated genes (Fig. 1), whereas a total of 163 GO biological pathway terms were significantly enriched in the upregulated genes. These terms were divided into 20 clusters, of which three were associated with cell growth and differentiation, responses to stimuli and apoptosis (Fig. 2).

### PPI network of the DEGs

**Figure 3:**
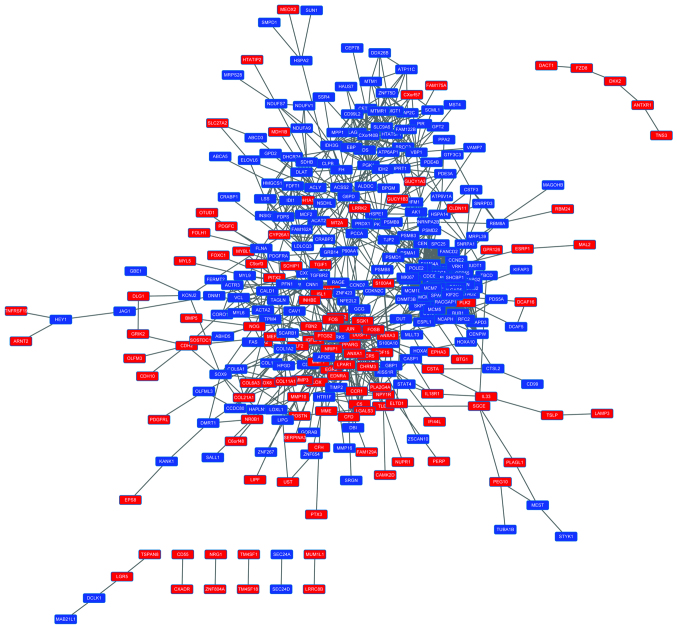
Protein-protein interaction network for the protein products of the differentially expressed genes. Upregulated genes are indicated in red and downregulated genes are indicated in blue. Links between the proteins indicate pairwise protein interactions.

**Figure 4:**
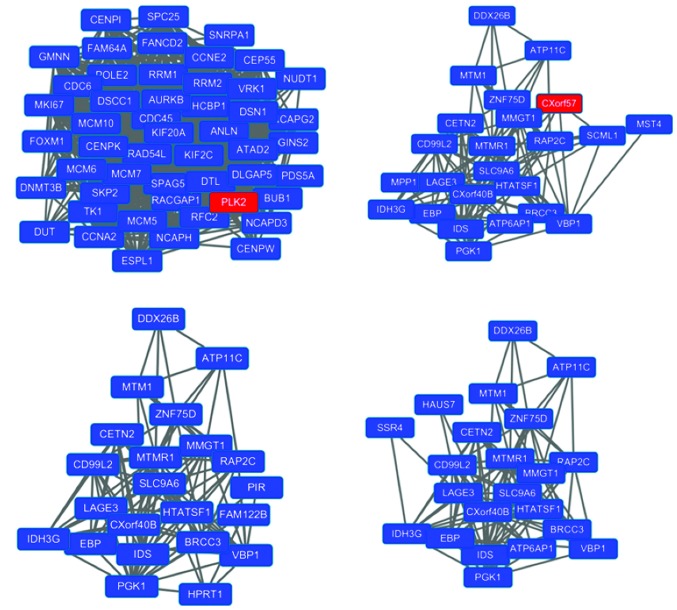
Top four subnetworks extracted from the entire network, which were selected according to the p-value rank in the ClusterONE analysis. Upregulated genes are indicated in red and downregulated genes are indicated in blue. (A) subnetwork 1; (B) subnetwork 2; (C) subnetwork 3; (D) subnetwork 1. Links between the proteins indicate pairwise protein interactions.

A PPI network consisting of 342 nodes was constructed for the DEGs (Fig. 3). A total of nine subnetworks were identified by ClusterONE (P<0.01). The top four subnetworks are shown in Fig. 4. Functional enrichment analysis indicated that subnetwork 1 (Fig. 4A) was predominantly associated with the cell cycle, subnetwork 2 (Fig. 4B) was associated with phosphoric acid metabolism and subnetwork 4 (Fig. 4D) was linked with the formation of central body and microtubules. They were all associated with cell division (Table II). No GO terms or pathways were enriched in subnetwork 3 (Fig. 4C).

### Association between enhancer of zeste homolog 2 (EZH2) and cisplatin-resistance in ovarian cancer

**Figure 5:**
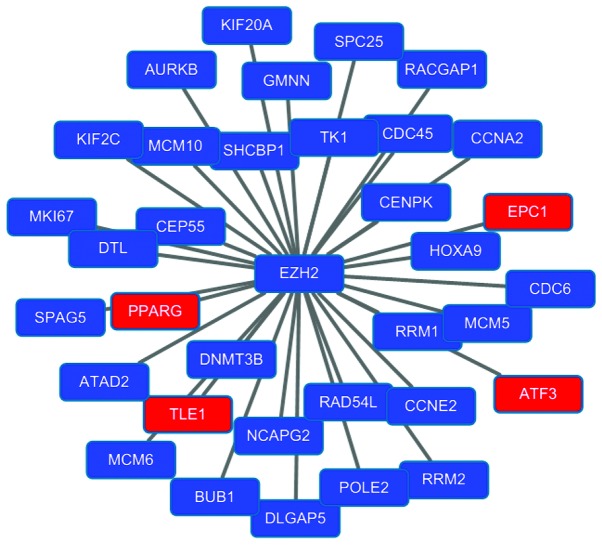
Differentially expressed genes interacting with enhancer of zeste homolog 2 (EZH2). Upregulated genes are in red, downregulated genes are in blue. Links between the proteins indicate pairwise protein interactions.

**Figure 6:**
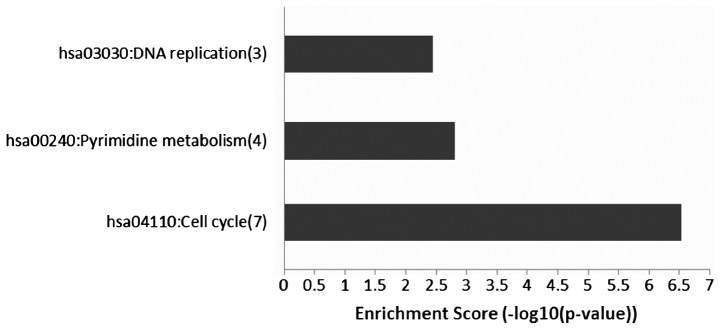
Kyoto Encyclopedia of Genes and Genome enrichment analysis identified three pathways significantly enriched in the differentially expressed genes interacting with enhancer of zeste homolog 2.

Previous studies have indicated that (EZH2) is involved in resistance of ovarian cancer cells to platinum-based drugs, such as cisplatin, carboplatin and paclitaxel (23,24). The present study found that EZH2 was down-regulated in cisplatin-sensitive cells (P=0.007; logFC=−0.35) and upregulated in cisplatin-resistant cells. A total of 34 DEGs (score ≥0.4) interacting with EZH2 were identified by STRING (Fig. 5). A total of three KEGG pathways were significantly enriched in the DEGs: DNA replication, pyrimidine metabolism and cell cycle (Fig. 6). Similar results were obtained in the GO enrichment analysis, in which 38 GO biological pathway terms were identified and divided into three clusters. Of these three clusters, two were associated with the cell cycle and the third was associated with DNA replication (Table III). These results suggested that EZH2 affected the cisplatin-resistance of ovarian cancer cells via modulation of the cell cycle.

## Discussion

In the present study, the gene expression profiles of cisplatin-sensitive ovarian cancer cells were compared with those of cisplatin-resistant ovarian cancer cells. A total of 556 DEGs were identified in the cisplatin-sensitive ovarian cancer cells, of which 246 were upregulated and 310 were downregulated. Functional enrichment analysis revealed that metabolism-associated pathways, DNA replication and the cell cycle were significantly enriched in the downregulated genes, while cell growth and differentiation, responses to stimuli and apoptosis were significantly enriched in the upregulated genes. These findings were in accordance with known biochemical mechanisms of cisplatin cytotoxicity (6,7,25,26). In addition, a PPI network, including 342 nodes, was constructed for the DEGs. Subnetworks linked to the cell cycle, phosphoric acid metabolism and formation of central body and microtubules were extracted from the entire network. These findings may assist in further elucidating the molecular mechanisms of cisplatin cytotoxicity and cisplatin resistance.

EZH2, a member of the polycomb-group family, is a specific histone 3 lysine 27 methylt ransferase, and is important in tumorigenesis and cancer progression through epigenetic gene silencing and chromatin remodeling (27). Hu *et al* reported that the overexpression of EZH2 contributes to acquired cisplatin resistance in ovarian cancer cells (28). Rizzo *et al* observed the EZH2 is overexpressed in ovarian cancer stem cell-like side populations and is associated with drug resistance (29). A similar role for EZH2 has been reported in lung cancer (30). The present study further investigated EZH2 and found that it was downregulated in cisplatin-sensitive ovarian cancer cells. A total of 34 DEGs directly interacting with EZH2 were identified. Functional enrichment analysis suggested that DNA replication, pyrimidine metabolism and cell cycle were significantly enriched in the 34 DEGs. Cyclin E2 (CCNE2) is involved in the cell cycle G1/S transition and it has been reported that the overexpression of CCNE2 is associated with endocrine resistance in human breast cancer cells (31,32). A study by Tu *et al* further indicated that the inhibition of CCNE2 can reduce tamoxifen resistance in breast cancer cells (33). Cyclin A2 (CCNA2) is also closely associated with tamoxifen resistance, as its expression is positively associated with genes overexpressed in endocrine therapy resistant samples (34). Minichromosome maintenance complex component 5 (MMC5) and MMC6, members of the minichromosome maintenance (MCM) family of chromatin-binding proteins, are essential for the initiation of eukaryotic genome replication. Gao *et al* suggested that genes involved in genome stability may contribute significantly to the development of camptothecins resistance in melanoma, with MCM5 as one of the candidates (35). The present study hypothesized that these genes may be involved in the cisplatin-resistance of ovarian cancer cells in a similar way. BUB1 mitotic checkpoint serine/threonine kinase (BUB1) not only regulates chromosome segregation (36), but also mediates cell death in response to chromosome missegregation (37). Overexpression of BUB1 contributes tothe cytogenetic and morphologic progression of clear cell kidney carcinomas (38). The present study demonstrated that it is upregulated in cisplatin-resistant ovarian cancer cells, suggesting it may be involved in the acquisition of drug resistance. These findings indicated that EZH2 may lead to drug resistance via regulation of the cell cycle.

In conclusion, the present study identified a number of DEGs in cisplatin-sensitive ovarian cancer cells, compared with cisplatin-resistant ovarian cancer cells. These findings may advance current understanding of the molecular mechanisms underlying cisplatin cytotoxicity and cisplatin resistance. EZH2 and its interactors were also identified, which may be used as targets to modulate drug resistance and thus benefit the treatment of ovarian cancer.

## Figures and Tables

**Table I:** Summary of the five cisplatin-sensitive and five cisplatin-resistant replicates, obtained from the Gene Expression Omnibus.

Accession	Description
GSM385721	Parental A2780 (cisplatin-sensitive), biological replicate 1
GSM385722	Parental A2780 (cisplatin-sensitive), biological replicate 2
GSM385723	Parental A2780 (cisplatin-sensitive), biological replicate3
GSM385724	Parental A2780 (cisplatin-sensitive), biological replicate 4
GSM385725	Parental A2780 (cisplatin-sensitive), biological replicate 5
GSM385726	Round5 A2780 (cisplatin-resistant), biological replicate 1
GSM385727	Round5 A2780 (cisplatin-resistant), biological replicate 2
GSM385728	Round5 A2780 (cisplatin-resistant), biological replicate 3
GSM385729	Round5 A2780 (cisplatin-resistant), biological replicate 4
GSM385730	Round5 A2780 (cisplatin-resistant), biological replicate 5

**Table II:** Functional enrichment analysis of the differentially expressed genes in the subnetworks.

Subnetwork	Term	P-value	Gene
1	GO:0007049: cell cycle	1.11E-23	FOXM1, ANLN, CEP55, AURKB, CCNE2, KIF2C, SPC25, CDC45, NCAPH, MCM7, NCAPG2, BUB1, CCNA2, CDC6, MKI67, PDS5A, DSN1, GMNN, DLGAP5, SKP2, ESPL1, RACGAP1, RAD54L, NCAPD3, MCM6, PLK2, SPAG5, FANCD2, DSCC1
	GO:0000279: M phase	2.85E-19	CDC6, MKI67, PDS5A, DSN1, DLGAP5, ANLN, ESPL1, AURKB, CEP55, RAD54L, NCAPD3, SPC25, KIF2C, NCAPH, NCAPG2, FANCD2, SPAG5, BUB1, CCNA2, DSCC1
	GO:0022403: cell cycle phase	8.57E-19	CDC6, MKI67, PDS5A, DSN1, DLGAP5, SKP2, ANLN, ESPL1, AURKB, CEP55, RAD54L, NCAPD3, SPC25, KIF2C, NCAPH, NCAPG2, FANCD2, SPAG5, BUB1, CCNA2, DSCC1
	GO:0007067: mitosis	8.67E-18	CDC6, PDS5A, DSN1, DLGAP5, ANLN, ESPL1, AURKB, CEP55, NCAPD3, SPC25, KIF2C, NCAPH, NCAPG2, SPAG5, BUB1, CCNA2, DSCC1
	GO:0000280: nuclear division	8.67E-18	CDC6, PDS5A, DSN1, DLGAP5, ANLN, ESPL1, AURKB, CEP55, NCAPD3, SPC25, KIF2C, NCAPH, NCAPG2, SPAG5, BUB1, CCNA2, DSCC1
	hsa04110: ell cycle	9.91E-11	CCNE2, CDC6, CDC45, MCM7, SKP2, BUB1, ESPL1, CCNA2, MCM5, MCM6
	hsa03030: DNA replication	6.04E-06	MCM7, POLE2, RFC2, MCM5, MCM6
	hsa00240: yrimidine metabolism	2.86E-04	POLE2, RRM2, RRM1, TK1, DUT
2	GO:0006793: phosphorus metabolic process	0.03	MTM1, MTMR1, ATP6AP1, PGK1, MST4
	GO:0006796: phosphate metabolic process	0.03	MTM1, MTMR1, ATP6AP1, PGK1, MST4
4	GO:0051297: centrosome organization	0.039	CETN2, HAUS7
	GO:0031023: microtubule organizing center organization	0.043	CETN2, HAUS7

**Table III:** Gene Ontology functional enrichment analysis of the genes interacting with enhancer of zeste homolog 2.

Term	P-value
Cluster 1	
GO:0007049: cell cycle	5.50E-12
GO:0000279: M phase	1.79E-10
GO:0051301: cell division	1.24E-09
GO:0000280: nuclear division	1.86E-09
GO:0007067: mitosis	1.86E-09
GO:0022403: cell cycle	2.04E-09
GO:0000087: M phase of mitotic cell cycle	2.18E-09
GO:0048285: organelle fission	2.64E-09
GO:0022402: cell cycle process	3.98E-09
GO:0000278: mitotic cell cycle	1.68E-07
Cluster 2	
GO:0000075: cell cycle checkpoint	6.75E-05
GO:0051726: regulation of cell cycle	1.30E-04
GO:0007346: regulation of mitotic cell cycle	0.006
GO:0031570: DNA integrity checkpoint	0.007
Cluster 3	
GO:0051726: regulation of cell cycle	1.30E-04
GO:0045934: negative regulation of nucleobase, nucleoside, nucleotide and nucleic acid metabolic process	0.001
GO:0051172: negative regulation of nitrogen compound metabolic process	0.001
GO:0010558: negative regulation of macromolecule biosynthetic process	0.002
GO:0008156: negative regulation of DNA replication	0.002
GO:0031327: negative regulation of cellular biosynthetic process	0.002
GO:0009890: negative regulation of biosynthetic process	0.002
GO:0051053: negative regulation of DNA metabolic process	0.004
GO:0010605: negative regulation of macromolecule metabolic process	0.008
GO:0006275: regulation of DNA replication	0.01
GO:0051052: regulation of DNA metabolic process	0.031
